# Health care utilization for common childhood illnesses in rural parts of Ethiopia: evidence from the 2016 Ethiopian demographic and health survey

**DOI:** 10.1186/s12889-019-6397-x

**Published:** 2019-01-14

**Authors:** Muluneh Alene, Leltework Yismaw, Yebelay Berelie, Bekalu Kassie

**Affiliations:** 1 Department of Statistics, Mizan-Tepi University, Teppi, Ethiopia; 2 Department of Public Health, Debre Markos University, Debre Markos, Ethiopia; 3 Department of Statistics, Debre Markos University, Debre Markos, Ethiopia; 4 Department of Midwifery, Debre Markos University, Debre Markos, Ethiopia

**Keywords:** Childhood illnesses, Healthcare seeking, Rural area, EDHS, Ethiopia

## Abstract

**Background:**

Generally, health care utilization in developing countries is low particularly rural community have lower health care utilization. Despite this fact, little is known about the magnitude and determinants of health care utilization for common childhood illnesses in Ethiopia. Thus, this study was conducted to determine the magnitude and to identify determinants of health care utilization for common childhood illnesses in the rural parts of Ethiopia.

**Methods:**

For this study, data were obtained from the 2016 Ethiopian demographic and health survey. A total of 1576 mothers of under-five children were included in the analysis. Data analysis was performed using R software. Both univariable and multivariable logistic regression analysis were fitted to identify the determinants of health care utilization. Variables with a 95% confidence interval for odds ratio excluding one were considered as significant determinants of the outcome.

**Results:**

The findings of this study revealed that only half (49.7%) (95%CI: 46.1–53.4%), 40.9% (95%CI 37.6–44.2%), and 38.0% (95%CI: 34.7–41.4%) of the children utilized health care for diarrhea, fever, and cough, respectively. Children age between 12 and 23 months (AOR: 1.58, 95%CI: 1.08–2.31), maternal education (AOR: 1.96, 95%CI: 1.34–2.88), and giving birth at health facilities (AOR: 1.49, 95%CI: 1.04–2.13) were found to be the determinants of health care utilization for diarrhea. Marital status (AOR: 0.25, 95%CI: 0.06–0.81), husbands’ education (AOR: 1.37, 95%CI: 1.01–1.86), and giving birth at health facilities (AOR: 1.51, 95%CI: 1.09–2.10) were factors significantly associated with health care utilization for fever. Children age between 12 and 23 months (AOR: 1.51, 95%CI: 1.03–2.22), maternal education (AOR: 1.70, 95%CI: 1.18–2.44), and giving birth at health facilities (AOR: 1.74, 95%CI: 1.23–2.46) were significantly associated with health care utilization for cough.

**Conclusions:**

Low health care utilization for childhood illnesses was noticed. The health care utilization for diarrhea and cough was lower for children of ages between 0 and 11 months, mothers without formal education and home-delivered children’s. The health care utilization for fever was lower for separated parents, husbands without formal education, giving birth at home and from the poorest family. Programs to improve the educational status of a household are essential for better care utilization and children development.

## Background

Globally, an estimated 5.9 million children died before the age of 5 years in 2015. South Asia and sub-Saharan Africa (SSA) countries contributed 31 and 50% of worldwide under-five deaths respectively [1]. Especially, SSA has the highest under-five mortality rate in the world. In this region, under-five mortality rate was estimated to be 79 per 1000 live births in 2016 [2]. Ethiopia is one of the SSA countries having a high rate of under-five mortality. In Ethiopia, according to the recent Ethiopian Demographic and Health Survey (EDHS, 2016) report, under-five mortality was 67 deaths per 1, 000 live births [3]. Evidence suggested that most of under-five deaths in low and middle-income countries are caused by easily treatable and preventable diseases such as; diarrhea, fever, cough, pneumonia, and malaria, despite the existing interventions [4, 5].

Despite health care utilization for childhood illnesses has a tremendous effect on the reduction of childhood mortality and morbidity, majority of children died before reaching to the health facilities mainly due to delays in seeking health care [6]. Different studies conducted elsewhere documented that distance from the health care facilities, poor knowledge about the symptoms of diseases, perceived in-curability of illness, lack of money, limited health care access, and a long period of waiting for medical services were the main reasons for low health care utilization in developing countries [7–10]. In developing countries, health care utilization and integrated management of childhood illnesses are generally low particularly rural community have lower rate of health care utilization [4, 11–15]. For example, a study conducted in North Shoa, Ethiopia revealed that less than half (43.2%) of the sick rural children utilized health care as compared to 87.2% of the sick urban children [13].

In the rural parts of Kenya, health care was sought for only one-third of the children with both fever and diarrhea illnesses [16], and 82% of the caregivers used health centers for treating diarrhea in rural Uganda [17]. Nearly three-quarters (74.3%) of the children with fever, 77.3% of the children with Acute Respiratory Infection (ARI) and 23.0% of the children with diarrhea were initially treated at a health facilities in rural Tanzania [18]. In rural Niger, the medical care was sought for 70.4% of under-five children with diarrhea illness [19]. Moreover, among under-five children, health care was sought for less than one-third (30%), 35, and 44% of children with ARI, fever, and diarrhea respectively in Ethiopia [20].

Decision making about the health care utilization for a sick child is not only influenced by mothers/caregivers effort, but it is also shared by others in the household and guided by how the symptoms were perceived [21]. Additionally, the health care utilization for an ill child was described by cultural, social, and community-based resources and familiarity to the biomedical and governmental health care providers [22]. Mothers/caregivers with a household of higher socioeconomic status and a child showing sever symptoms of illnesses were more likely to seek health care for childhood illnesses [4, 12, 13, 16, 18, 23–26]. Furthermore, recognizing the severity and types of reported illnesses were associated with mothers/caregivers health care utilization for childhood illnesses [4, 11, 13, 27, 28].

Despite proper health care utilization reduces the morbidity and mortality of children resulted from easily treatable and preventable childhood illnesses, little is known about the magnitude and determinants of health care utilization for common childhood illnesses in Ethiopia, particularly in rural areas. Thus, this study was conducted to determine the magnitude and to identify the determinants of health care utilization for common childhood illnesses (diarrhea, fever, and cough) in the rural parts of Ethiopia. The findings of this study will inform program planers and decision makers working in the area of child health. Moreover, this study could help to evaluate the Sustainable Development Goals (SDGs) which, aimed to reduce under-five mortality rate to 25 deaths per 1000 live births or below and ending preventable childhood deaths in 2030 [29].

## Methods

### Study setting and data description

Geographically, Ethiopia has nine geographical regions and two administrative cities. Each region has urban and rural kebeles (lowest administrative unit). More than 80 % (80%) of the population are living in three regions; namely Oromia, Amhara, and Southern Nations, Nationalities, and Peoples’ Region (SNNPR). About 84% of the total population are living in the rural parts of the country. The highest proportion (90%) of rural residents are from SNNPR whereas, the lowest proportion (32.5%) of rural residents are from Dire-Dewa [30].

The data for the present study were obtained from the 2016 EDHS, which is the fourth demographic and health survey conducted in Ethiopia. It was executed by Central Statistics Agency (CSA) in collaboration with Federal Ministry of Health (FMOH) and the Ethiopian Public Health Institute (EPHI). The technical assistance and funding were supported by the International Classification of Functioning (ICF) and development partners. Accordingly, the data were collected from January 18, 2016, to June 27, 2016, which provided a wide range overview of population, particularly maternal and child health issues. This survey was also conducted with the aim of estimating the demographic and health indicators. The sampling frame used for EDHS was the Ethiopian Population and Housing Census (PHC), which was conducted in 2007 by CSA. The PHC frame provides a complete list of 84,915 enumeration areas (EAs), and each EA covers on average 181 households. The 2016 EDHS sample was stratified and selected using two stages. In the first stage, a total of 645 EAs (202 from urban areas and 443 from rural areas) were selected independently with probability proportional to EA size. Then, a household listing operation was carried out in all of the selected EAs, and the resulting lists of households were served as a sampling frame for the selection of households in the second stage. In the second stage, a fixed number of 28 households per cluster were selected with an equal probability systematic selection from the newly created household listing. All reproductive age women (age 15–49 year) and who were either permanent residents of the selected households or visitors, or who stayed in the household the night before the survey were eligible to be interviewed. Based on fertility of women this age range has been selected.

### Data extraction and study population

**Fig. 1:**
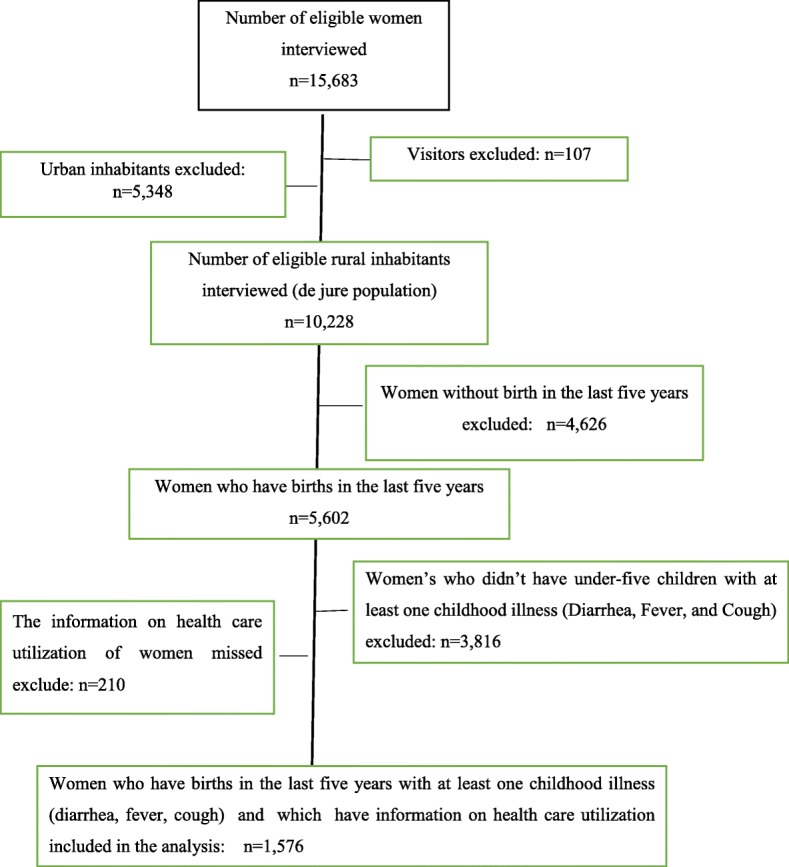
Diagrammatic presentation of data extraction from the 2016 Ethiopian Demographic and Health survey

The 2016 EDHS interviewed a total 15,683 women between ages of 15–49 year. Of these, 10,335 (66%) were from rural areas. The data for the present study were extracted as follows: First, women who gave births in the last 5 years were identified. Next, children with diarrhea and/or fever, and/or a cough in the 2 weeks preceding the survey period were identified. Finally, a total of 1576 rural mothers who had at least one under-five children with common childhood illness (diarrhea, fever, and cough) were included. It is customary to get more than one under-five children per a household. However, for data quality, if there were more than one under-five children per household, data were collected from children with the last (recent) birth. Whereas, women who didn’t give a birth in the last 5 years, women who hadn’t under-five children with at least one childhood illness, and the information on which the health care utilization of mother for childhood illnesses was missed were excluded from the study. The details of data extraction to obtain the study population is presented in Fig. 1.

### Variables and measurements

The study has three main outcome variables. These include the health care utilization for childhood diarrhea, fever, and cough. The 2016 EDHS collected data whether the child had diarrhea and/or fever, and/or cough in the 2 weeks preceding the survey. The health care utilization for childhood illnesses was also assessed by interviewing the mothers. The mothers were considered as they sought health center if they seek medical treatment from a defined governmental or non-governmental health facilities for childhood illnesses.

Child-related variables (age of child, sex of child, birth order of a child and place of delivery), parental related variables (mothers and fathers education level, age and marital status of mother), and household characteristics (household wealth index, total under-five births and exposure to mass media) were used to assess the health care utilization for common childhood illness. Household wealth indexes were determined by scores. Scores were computed using a principal component analysis, which considers the number, and kinds of consumer goods they own, housing characteristics, and flooring materials. National wealth quintiles were compiled by assigning the household score to each household member, ranking each person in the household population by her or his score, and then dividing the distribution into five equal categories.

### Data processing and analysis

The extracted data were cleaned, coded, and analyzed using “R” version 3.5 statistical software. We used both descriptive (percentage and frequency tables) and inferential statistics for data presentation. The investigators applied both the univariable and multivariable binary logistic regression analysis to identify the determinant factors of health care utilization for common childhood illnesses. Both Crude Odds Ratio (COR) and Adjusted Odds Ratio (AOR) with 95% confidence interval (CI) were computed to measure the associations and to identify the significant determinants. Variables which have a 95% confidence interval for odds ratio excluding one were considered as a significant determinants of health care utilization for common childhood illnesses (diarrhea, fever, and cough).

## Results

### Descriptive results of health care utilization for childhood illnesses among included variables

In the study, a total of 1576 rural mothers who had under-five children were involved. From a total of under-five children included in this study, 806, 851, and 730 of the children had cough, fever, and diarrhea respectively. However, only half (49.7%) of the children with diarrhea, 40.9% of the children with fever, and 38.0% of the children with cough were sought for health care (Table 1). Of the respondents, only 472 (29.9%) of them have formal (primary and above) education. More than half (55%) of the mothers age were less than 30 years, and the majority (94%) were married. About one-third 516 (32.7%) and 373 (23.7%) of them want to have no more children and can access mass media respectively. From the involved under-five children, nearly half 732 (46.4%) of them were females.

**Table 1:** Magnitude of health care utilization for common childhood illnesses (diarrhea, fever and cough) in rural area of Ethiopia from the 2016 Ethiopian Demographic and Health survey

Types of illness	Magnitude of health care utilization (95%CI)
Diarrhea (*n* = 730)	49.7% [46.1–53.4]
Fever (*n* = 851)	40.9% [37.6–44.2]
Cough (*n* = 806)	38.0%[34.7–41.4]

Table 2 shows the descriptive results of the mother’s health care utilization for each childhood illness among the included variables. Nearly half 195 (49.2%), 181 (40%), and 149 (36.4%) of female children with diarrhea, fever, and cough respectively were sought for medical care. Among the respondents who have primary and above education level, about 152 (63.6%), 129 (48.9%), and 119 (49%) of them were seeking medical care for diarrhea, fever, and cough respectively.

**Table 2:** Descriptive results of health care utilization for common childhood illnesses (diarrhea, fever and cough) among variables from the 2016 Ethiopian Demographic and Health Survey

Variables	Care-seeking for diarrhea illness (%)	Total	Care-seeking for fever illness (%)	Total	Care-seeking for cough illness (%)	Total
Child age						
0–11 month	104 (47.3%)	222	102 (40.5%)	252	96 (38.4%)	250
12–23 month	146 (56.4%)	259	125 (45.0%)	278	108 (45%)	240
24–35 month	75 (51.0%)	147	74 (42.8%)	173	68 (36.8%)	185
36–47 month	23 (34.3%)	67	26 (31.3%)	83	20 (26.3%)	76
48–59 month	15 (40.5%)	37	21 (32.3%)	65	14 (25.5%)	55
Sex of child						
Male	195 (49.2%)	396	181 (40.0%)	452	149 (36.4%)	409
female	168 (50.3%)	334	167 (41.9%)	399	157 (39.5%)	397
Birth order						
First birth	73 (54.1%)	135	70 (42.9%)	163	62 (39.7%)	156
Second birth	57 (57.6%)	99	48 (42.1%)	114	37 (36.6%)	101
Third birth	55 (50.0%)	110	43 (40.6%)	106	42 (40.0%)	105
Forth birth and above	178 (46.1%)	386	187 (40.0%)	468	165 (37.2%)	444
Total under-five birth						
One birth	196 (49.7%)	394	186 (38.6%)	482	156 (34.7%)	450
Two births	142 (49.8%)	285	137 (44.3%)	309	127 (42.9%)	296
Three and above births	25 (49.0%)	51	25 (41.7%)	60	23 (38.3%)	60
Marital status of mothers						
Married	348 (51.0%)	683	330 (42.1%)	783	293 (39.0%)	752
Divorced	12 (38.7%)	31	12 (29.3%)	41	9 (27.3%)	33
Widowed	2 (22.2%)	9	3 (37.5%)	8	1 (33.3%)	3
Separated	1 (14.3%)	7	3 (15.8%)	19	3 (16.7%)	18
Mothers education						
No formal education	211 (43.0%)	491	219 (37.3%)	587	187 (33.2%)	563
Primary and above	152 (63.6%)	239	129 (48.9%)	264	119 (49.0%)	243
Mothers age						
Less than 30 years	220 (54.5%)	404	195 (43.3%)	450	180 (40.7%)	442
30 years and above	143 (43.9%)	326	153 (38.2%)	401	126 (34.6%)	364
Husbands education						
No formal education	157 (44.4%)	354	142 (34.7%)	409	132 (33.1%)	399
Primary and above	206 (54.8%)	376	206 (46.6%)	442	174 (42.8%)	407
Desire to have more child						
Wants more children	256 (52.2%)	490	244 (42.8%)	570	219 (40.3%)	544
Wants no more children	107 (44.6%)	240	104 (37.0%)	281	87 (33.2%)	262
Wealth index						
Poorest	119 (43.9%)	271	114 (32.2%)	343	106 (35.5%)	299
Poorer	73 (47.1%)	155	76 (43.2%)	176	67 (36.4%)	184
Middle	74 (53.2%)	139	65 (44.5%)	146	52 (35.9%)	145
Richer	73 (58.4%)	125	75 (53.2%)	141	67 (50.0%)	134
Richest	24 (60.0%)	40	18 (40.0%)	45	14 (31.8%)	44
Exposure to mass media						
No	269 (48.2%)	558	260 (40.2%)	647	221 (36.8%)	600
Yes	94 (54.7%)	172	88 (43.1%)	204	85 (41.3%)	206
Place of delivery						
Home	228 (45.5%)	501	219 (36.7%)	596	191 (33.7%)	567
Health facility	135 (59.0%)	229	129 (50.6%)	255	115 (48.1%)	239

The finding of this study also indicated that among respondents who had opportunities to access mass media for health education, about 94 (54.7%), 88 (43.1%), and 85 (41.3%) of the participants were seeking medical care for diarrhea, fever, and cough respectively. Among respondents who want to have no more children, less than half 107 (44.6%), 104 (37.0%), and 87 (33.2%) of them were seeking medical care for diarrhea, fever, and cough respectively. Moreover, among households who had two under-five births, nearly half 142 (49.8%), 137 (44.3%), and 127 (42.9%) of under-five children with diarrhea, fever, and cough were seeking medical care respectively. Similarly, among households who had three and above under-five births, nearly half 25 (49.0%), 25 (41.7%) and 23 (38.3%) of under-five children with diarrhea, fever, and cough were seeking medical care respectively.

### Determinants of health care utilization for diarrhea

Multivariable binary logistic regression analysis was conducted to determine the health care utilization for diarrheal illness. Accordingly; child age, maternal education, and place of delivery were factors significantly associated with health care utilization for diarrheal illness. The odds of health care utilization for diarrheal illness among children between the age of 12 and 23 months was 1.58 (AOR: 1.58, 95%CI: 1.08–2.31) times higher as compared to children whose age were between 0 and 11 months. The odds of health care utilization for diarrheal illness among mothers who had formal (primary and above) education was 1.96 (AOR: 1.96, 95%CI: 1.34–2.88) times higher as compared to mothers without formal education. Moreover, the odds of health care utilization among mothers who gave birth at the health care facilities was 1.49 (AOR: 1.49, 95%CI: 1.04–2.13) times higher as compared to mothers who gave birth at home (Table 3).

**Table 3:** Determinants of health care utilization for diarrhea illness from the 2016 Ethiopian Demographic and Health survey (*n* = 730)

Variables	Health care utilization		COR(95%CI)	AOR(95%CI)
	Sought health center	Not -sought health center		
Child age in months				
0–11 month	104	116	Ref.	Ref.
12–23 month	146	113	1.44 (1.01–2.07)	1.58 (1.08–2.31)^*^
24–35 month	75	72	1.16 (0.77–1.77)	1.38(0.88–2.17)
36–47 month	23	44	0.58 (0.33–1.02)	0.69(0.36–1.28)
48–59 month	15	22	0.76(0.37–1.53)	0.86–0.39-1.87)
Sex of child				
Male	195	201	Ref.	Ref.
female	168	166	1.04 (0.78–1.39)	1.03 (0.75–1.40)
Birth order of child				
First birth	73	62	Ref.	Ref.
Second birth	55	42	1.15(0.68–1.95)	1.46(0.78–2.72)
Third birth	53	55	0.85 (0.51–1.41)	1.27(0.69–2.35)
Forth birth and above	178	208	0.73 (0.49–1.08)	1.32 (0.71–2.48)
Total under-five birth				
One birth	196	198	Ref.	
Two births	142	143	1.00(0.74–1.36)	0.88 (0.59–1.31)
Three and above births	25	26	0.97 (0.54–1.74)	0.98 (0.49–1.93)
Mothers marital status				
Married	348	335	Ref.	Ref.
Divorced	12	19	0.61 (0.28–1.26)	0.50 (0.21–1.12)
Widowed	2	7	0.28(0.04–1.15)	0.34 (0.05–1.49)
Separated	1	6	0.16 (0.01–0.95)	0.13 (0.01–0.87)
Mothers education level				
No formal education	211	280	Ref.	Ref.
Primary and above	152	87	2.32(1.69–3.20)	1.96 (1.34–2.88)^***^
Mothers age				
Less than 30 years	220	184	Ref.	Ref.
30 years and above	143	183	0.65 (0.49–0.88)	0.84(0.55–1.29)
Husbands education level				
No formal education	157	197	Ref.	Ref.
Primary and above	206	170	1.52(1.14–2.04)	1.11(0.80–1.55)
Desire to have more child				
Wants more children	256	234	Ref.	Ref.
Wants no more children	107	133	0.74 (0.54–1.00)	0.79(0.56–1.12)
Wealth index				
Poorest	119	152	Ref.	Ref.
Poorer	73	82	1.14 (0.76–1.69)	1.05(0.69–1.61)
Middle	74	65	1.45(0.97–2.20)	1.17 (0.74–1.83)
Richer	73	52	1.79 (1.17–2.76)	1.25(0.76–2.05)
Richest	24	16	1.92 (0.98–3.83)	1.19(0.56–2.60)
Exposure to mass media				
No	269	289	Ref.	Ref.
Yes	94	78	1.29 (0.92–1.80)	1.18 (0.80–1.74)
Place of delivery				
Home	228	273	Ref.	Ref.
Health facility	135	94	1.72 (1.25–2.36)	1.49(1.04–2.13)^*^

Ref. =Reference category

^***^ *p < 0.001;* ^**^ *p < 0.01;* ^*^ *p < 0.05*

### Determinants of health care utilization for fever

In multivariable logistic regression analysis, mother’s marital status, husbands educational level, wealth index, and place of delivery were found to be significant determinants of health care utilization for fever. Mothers who are living separately (apart) from their husband (AOR: 0.25, 95%CI: 0.06–0.81) were less likely to seek health care for fever as compared to married mothers (living together). A husband who had formal (primary and above) education were 1.37 (AOR: 1.37, 95%CI: 1.01–1.86) times more likely to seek medical care as compared to who hadn’t formal education. Moreover, mother who gave birth at health facilities were 1.5 (AOR: 1.51, 95%CI: 1.09–2.10) times more likely to seek health care for fever as compared to mothers who gave birth at home. Furthermore, respondents from households with the poorer (AOR: 1.53, 95%CI: 1.03–2.27), middle (AOR: 1.60, 95%CI: 1.04–2.44), and richer (AOR: 2.00, 95%CI: 1.27–3.17) wealth status were more likely to seek health care for fever as compared to respondents from households with the poorest wealth status (Table 4).

**Table 4:** Determinants of health care utilization for fever illness from the 2016 Ethiopian Demographic and Health survey (*n* = 851)

Variables	Health care utilization		COR(95%CI)	AOR(95%CI)
	Sought health center	Not-sought health center		
Child age in months				
0–11 month	102	150	Ref.	Ref.
12–23 month	125	153	1.20(0.85–1.69)	1.37 (0.95–1.97)
24–35 month	74	99	1.10 (0.74–1.63)	1.44 (0.94–2.21)
36–47 month	26	57	0.67 (0.39–1.13)	0.89 (0.49–1.59)
48–59 month	21	44	0.70 (0.39–1.24)	1.05 (0.54–2.00)
Sex of child				
Male	181	271	Ref.	Ref.
female	167	232	1.08 (0.82–1.42)	1.08 (0.82–1.44)
Birth order of child				
First birth	70	93	Ref.	Ref.
Second birth	48	66	0.97(0.59–1.57)	0.87 (0.49–1.51)
Third birth	43	63	0.91(0.55–1.49)	0.76 (0.42–1.37)
Forth birth and above	187	281	0.88 (0.62–1.27)	0.96(0.55–1.67)
Total under-five birth				
One birth	186	296	Ref.	Ref.
Two births	137	172	1.27(0.95–1.69)	1.33(0.92–1.92)
Three and above births	25	35	1.14 (0.65–1.95)	1.43(0.75–2.68)
Mothers marital status				
Married	330	453	Ref.	Ref.
Divorced	12	29	0.57 (0.28–1.10)	0.59 (0.27–1.21)
Widowed	3	5	0.82 (0.17–3.38)	0.94(0.18–4.05)
Separated	3	16	0.26 (0.06–0.78)	0.25 (0.06–0.81)^*^
Mothers education level				
No formal education	219	368	Ref.	Ref.
Primary and above	129	135	1.61 (1.20–2.15)	1.26 (0.89–1.77)
Mothers age				
Less than 30 years	195	255	Ref.	Ref.
30 years and above	153	248	0.81 (0.61–1.06)	0.89(0.61–1.32)
Husbands education level				
No formal education	142	267	Ref.	Ref.
Primary and above	206	236	1.64 (1.25–2.17)	1.37 (1.01–1.86)^*^
Desire to have more child				
Wants more children	244	326	Ref.	Ref.
Wants no more children	104	177	0.79 (0.58–1.05)	0.81 (0.58–1.12)
Wealth index				
Poorest	114	229	Ref.	Ref.
Poorer	76	100	1.53 (1.05–2.22)	1.53 (1.03–2.27)^*^
Middle	65	81	1.61 (1.08–2.40)	1.60 (1.04–2.44)^*^
Richer	75	66	2.28 (1.53–3.41)	2.00 (1.27–3.17)^**^
Richest	18	27	1.34 (0.70–2.52)	1.15 (0.56–2.30)
Exposure to mass media				
No	260	387	Ref.	Ref.
Yes	88	116	1.13 (0.82–1.55)	0.90(0.63–1.29)
Place of delivery				
Home	219	377	Ref.	Ref.
Health facility	129	126	1.76 (1.31–2.37)	1.51(1.09–2.10)^*^

Ref. =Reference category

^**^ *p < 0.01;* ^*^ *p < 0.05*

### Determinants of health care utilization for cough

From the multivariable binary logistic regression analysis, child age, maternal educational status, and place of delivery were factors significantly associated with the health care utilization for cough. Children whose age are between 12 and 23 months (AOR: 1.51, 95%CI: 1.03–2.22) were 1.5 times more likely to seek medical care for cough as compared to children’s aged between 0 and 11 months. Furthermore, mothers who had formal (primary and above) education (AOR: 1.70, 95%CI: 1.18–2.44) were 1.7 times to seek health care for cough as compared who hadn’t formal education. Moreover, mothers who gave birth at health facility (AOR: 1.74, 95%CI:1.23–2.46) were 1.74 times more likely to seek health care for cough as compared to those giving birth at home (Table 5).

**Table 5:** Determinants of health care utilization for cough illness from the 2016 Ethiopian Demographic and Health survey (*n* = 806)

Variables	Health care utilization		COR(95%CI)	AOR(95%CI)
	Sought health center	Not-sought health center		
Child age in months				
0–11 month	96	154	Ref.	Ref.
12–23 month	108	132	1.31(0.92–1.88)	1.51(1.03–2.22)^*^
24–35 month	68	117	0.93(0.63–1.38)	1.21(0.79–1.87)
36–47 month	20	56	0.58(0.32–1.00)	0.85 (0.47–1.58)
48–59 month	14	41	0.55(0.28–1.04)	0.84 (0.40–1.73)
Sex of child				
Male	149	260	Ref.	Ref.
female	157	240	1.14(0.86–1.52)	1.19 (0.88–1.61)
Birth order of child				
First birth	62	94	Ref.	Ref.
Second birth	67	64	0.88 (0.52–1.47)	0.99(0.53–1.78)
Third birth	42	63	1.01(0.61–1.67)	1.09 (0.60–1.97)
Forth birth and above	165	279	0.89 (0.62–1.31)	1.34(0.75–2.43)
Total under-five birth				
One birth	156	294	Ref.	Ref.
Two births	127	169	1.42(1.05–1.91)	1.36(0.92–2.02)
Three and above births	23	37	1.17 (0.66–2.03)	1.15 (0.59–2.21)
Mothers marital status				
Married	293	459	Ref.	Ref.
Divorced	9	24	0.59 (0.26–1.24)	0.72 (0.30–1.63)
Widowed	1	2	0.78(0.04–8.21)	1.21 (0.05–13.57)
Separated	3	15	0.31(0.07–0.96)	0.38 (0.09–1.24)
Mothers education level				
No formal education	187	376	Ref.	Ref.
Primary and above	119	124	1.92(1.42–2.62)	1.70 (1.18–2.44)^**^
Mothers age				
Less than 30 years	180	262	Ref.	Ref.
30 years and above	126	238	0.77(0.58–1.03)	0.83(0.55–1.25)
Husbands education level				
No formal education	132	267	Ref.	Ref.
Primary and above	174	233	1.51 (1.16–2.01)	1.22(0.89–1.69)
Desire to have more child				
Wants more children	219	325	Ref.	Ref.
Wants no more children	87	175	0.74 (0.54–1.00)	0.75 (0.53–1.06)
Wealth index				
Poorest	106	193	Ref.	Ref.
Poorer	67	117	1.04(0.71–1.53)	1.03 (0.68–1.55)
Middle	52	93	1.02 (0.67–1.54)	0.98 (0.62–1.54)
Richer	67	67	1.82 (1.20–2.76)	1.51(0.93–2.45)
Richest	14	30	0.85 (0.42–1.65)	0.56 (0.26–1.17)
Exposure to mass media				
No	221	379	Ref.	Ref.
Yes	85	121	1.20(0.87–1.66)	1.02 (0.71–1.48)
Place of delivery				
Home	191	376	Ref.	Ref.
Health facility	115	124	1.83 (1.34–2.48)	1.74(1.23–2.46)^**^

Ref. =Reference category

^**^ *p < 0.01;* ^*^ *p < 0.05*

## Discussion

The present study was conducted to determine the magnitude and to identify the determinant factors of health care utilization for common childhood illness in the rural parts of Ethiopia. The results of this study showed that nearly half (49.7%) (95%CI: 46.1–53.4%) of the children with diarrhea, 40.9% (95%CI: 37.6–44.2%) of the children with fever, and 38.0% (95%CI: 34.7–41.4) of the children with cough utilized health care from health facilities.

In this study, the health care utilization for diarrheal illness was higher as compared to fever and cough. The possible explanation for this variation might be due to the fact that commonly the rural community perceived diarrhea illnesses are more severe than fever and cough related illnesses. The observations of our study indicated that health care utilization for diarrhea illness was in agreement with a study conducted in the rural parts of South Africa (50%) [31]. On the other hand, our finding is lower than previous studies conducted in the rural parts of Niger (70.4%) and Uganda (76%) [17, 19]. The finding of the study also showed that health care utilization for fever illness was higher than a study conducted in Nigeria [32]. On the contrary, lower than a study conducted in a rural area of Tanzania [18]. The above inconsistent results might be due to the difference in socioeconomic status of the study participants [33]. Peoples standard of living is a vital factor for health and diseases related outcomes [34].

Child age, maternal educational status, and place of delivery were factors significantly associated with the health care utilization of childhood diarrheal illness. Respondent’s marital status, husband educational level, wealth status of a household, and place of delivery were also significantly associated with the health care utilization for fever illness. Furthermore, child age, maternal educational level, and place of delivery were significantly associated with the health care utilization for cough.

In the present study, mothers who had formal education (primary and above) were more likely to visit health facilities for medical care for a child with diarrheal illness as compared to mothers who hadn’t formal education. This finding is consistent with previous studies conducted in the rural areas of Bangladesh and Tanzania [18, 26, 35]. It is well known that education affects the health care utilization of the community. Therefore, educated mothers/caregivers can easily understand the severity of illnesses and seek health care within a short period of time. Additionally, mothers whose husband had formal education were more likely to seek health care for children with fever. This result is comparable with previous studies conducted in Ethiopia [11, 25]. This might be attributed to the fact that educated husbands could have a better understanding and knowledge towards childhood illnesses which in turn motivates their spouses to seek health care for their sick child.

The present study also revealed that mothers from households having the richer, middle, and poorer wealth status were more likely to seek medical treatment for fever as compared to the poorest household counterparts. This finding is in agreement with previous studies reported from Nepal and Ethiopia [25, 36]. Studies documented that the financial capability is one of the determinant factors which, highly influences the health care utilization for childhood illnesses [26, 37].

In this study, we observed that health care utilization for diarrhea and cough illnesses was higher among children between the age of 12 and 23 months as compared to children between the age of 0 and 11 months. This finding is consistent with a study conducted in the northwest, Ethiopia [11]. The possible explanation for this result might be in tradition emphasis is given for higher age of children in the rural community. The finding of this study showed that married mothers (living together) were more likely to seek health care for fever illness as compared to mothers who are living separately (apart) from their husband. In Oromia regional state of Ethiopia [27], care seeking for childhood illnesses was higher for married caregivers than those who were not currently married. It mainly due to caring for a child separately is difficult for social and financial reasons.

The present study also indicated that respondents who were giving birth at a health facility were more likely to seek health care for diarrhea and fever childhood illnesses as compared to mothers who were giving births at home. The possible explanation for this result might be following antenatal care (ANC) enable mothers to be aware of the advantages of seeking a health care at the time of child illness.

### Limitations of the study

Though the investigators did their best to indicate the magnitude and determinants of health care utilization for common childhood illness, it is not free from limitations. The nature of data limits the inclusion of all possible factors that could affect the health care utilization of mothers for childhood illnesses. Variables such as distance to health facilities and perceived severity were some of the plausible factors that were not measured in the present study. Measurement of childhood illnesses was taken based on the mother’s perception, and was not validated clinically.

## Conclusions

Low health care utilization of mothers for common childhood illnesses was noticed. The health care utilization for both diarrhea and cough illnesses was lower for children aged between 0 and 11 months, mothers without formal education and giving birth at home. The health care utilization for fever illness was lower for mothers living separately (apart) from their husband, mothers whose husband hadn’t formal education, giving birth at home and from the poorest family. Programs to improve the education level of a household are essential for better health care and children’s development. Providing health education and counseling to mothers in the study setting are the immediate response to the concerned body.

## Abbreviations

ANC: Antenatal care
AOR: Adjusted odds ratio
CI: Confidence interval
COR: Crude odds ratio
CSA: Central statistics agency
EA: Enumeration areas
EDHS: Ethiopian demographic and health survey
EPHI: Ethiopian public health institute
FMoH: Federal ministry of health
ICF: International classification of functioning, disability and health
PHC: Population and housing census
SSA: Sub-Saharan Africa
